# Evolutionary Dynamics in Structured Populations Under Strong Population Genetic Forces

**DOI:** 10.1534/g3.119.400605

**Published:** 2019-08-28

**Authors:** Alison F. Feder, Pleuni S. Pennings, Joachim Hermisson, Dmitri A. Petrov

**Affiliations:** * Department of Biology, Stanford University,; † Department of Integrative Biology, University of California Berkeley,; ‡ Department of Biology, San Francisco State University, and; § Faculty of Mathematics, University of Vienna

**Keywords:** approximate Bayesian computation, FST, Migration, Rapid adaptation, Simian-HIV, Spatial structure

## Abstract

In the long-term neutral equilibrium, high rates of migration between subpopulations result in little population differentiation. However, in the short-term, even very abundant migration may not be enough for subpopulations to equilibrate immediately. In this study, we investigate dynamical patterns of short-term population differentiation in adapting populations via stochastic and analytical modeling through time. We characterize a regime in which selection and migration interact to create non-monotonic patterns of population differentiation over time when migration is weaker than selection, but stronger than drift. We demonstrate how these patterns can be leveraged to estimate high migration rates using approximate Bayesian computation. We apply this approach to estimate fast migration in a rapidly adapting intra-host Simian-HIV population sampled from different anatomical locations. We find differences in estimated migration rates between different compartments, even though all are above $N_{e}m$ = 1. This work demonstrates how studying demographic processes on the timescale of selective sweeps illuminates processes too fast to leave signatures on neutral timescales.

A population’s structure in physical space can profoundly impact how it evolves. Structure can speed up or slow down evolution (Barton 1993; Whitlock 2003; Martens *et al.* 2011) and cause populations to reach evolutionary outcomes unlikely or impossible in well-mixed populations (Wright 1982; McLaren 2016; Bitbol and Schwab 2014), including the maintenance of genetic diversity (Karlin 1976; Allman and Weissman 2018) and the prevalence of cooperation (Hauert and Doebeli 2004), clonal interference (Martens and Hallatschek 2011) and speciation (Coyne 1992). Understanding population structure aids our interpretation of genetic data sampled from around the globe (Bradburd *et al.* 2016; Novembre *et al.* 2008; Berg *et al.* 2019). For these reasons, understanding and quantifying population structure has long been a goal of population biology.

Despite population structure’s importance, classical population genetics results suggest that even little migration among subpopulations destroys much of the genetic evidence of its existence. For example, under neutral evolution, long-term population differentiation is a function of the product of effective population size ($N_{e}$) and migration rate per individual in the population (*m*). Specifically, Wright showed that population differentiation measure $F_{ST}$ is equal to $1/\left(1+4N_{e}m\right)$ (Wright 1949) in an island model under neutral equilibrium. Therefore we might expect that when $N_{e}m$ is much smaller than one, local drift overwhelms migration and $F_{ST}$ is substantially elevated above zero (with up to a maximum value of one under certain conditions) and populations appear spatially structured. However, when $N_{e}m$ is much larger than one, migration is effectively much faster than drift, $F_{ST}$ approaches zero and the populations appear completely well-mixed. Although the island model framework has been criticized (Whitlock *et al.* 1999), the result is a population genetics classic and underlies all population genetics theory (and subsequent applications to data) under the modeling assumption of panmixia. Examples include detecting barriers to gene-flow by using patterns of local divergence to deduce locally reduced levels of genetic exchange.

Importantly, Wright’s equation describes population equilibrium. In this equilibrium, even very few migrants can mix a large population if gene flow occurs over timescales of $N_{e}$ generations. However, a different picture emerges over shorter timescales (*i.e.*, less than $N_{e}$ generations) where ecological processes can have considerable non-equilibrium effects. For example, an ecological disturbance in a population can drastically and immediately perturb allele frequencies in different demes. The duration and magnitude of this departure from equilibrium will depend on the specifics of the disturbance, and the rate at which migration returns the populations to equilibrium.

Non-instantaneous (but still rapid) perturbations in allele frequency that occur on a timescale much shorter than $1/N_{e}m$ may also leave transient patterns in population differentiation. Strong positive selection represents one such process. Importantly, this holds not only for heterogeneous selection (which will lead to permanent differentiation patterns), but also for spatially homogeneous selection, which we will focus on here.

Most straightforwardly, non-equilibrium population dynamics present a problem for interpreting population differentiation statistics, such as $F_{ST}$. Fast processes lead to signals of isolation which can be misinterpreted as weak migration or local adaptation preventing gene flow (Whitlock 2003). However, this also presents an opportunity: with dynamical data it may be possible to track non-equilibrium behavior of population differentiation over time after a strong perturbation to estimate migration.

In this paper, we develop intuitions about subdivided populations adapting rapidly to a new selective pressure and connected to each other by migration over short timescales. Although our theory is more general, we start with a concrete example of how Simian-HIV evolves drug resistance across multiple tissues. This example demonstrates how strong selection, abundant mutation and fast migration can interact to create patterns of substantial yet transient population differentiation over short, clinically relevant timescales. We investigate the parameter regime where these forces are jointly strong and find that when migration is significantly faster than drift but slower than selection, characteristic, non-monotonic patterns of population differentiation with respect to time emerge. As a proof of concept that such dynamical patterns might be leveraged for estimation, we present an approximate Bayesian computation (ABC) approach that exploits population differentiation over time to estimate migration rates far above levels possible to determine with drifting alleles over long timescales. Finally, we return to the Simian-HIV data and estimate between tissue migration rates unresolvable in the neutral equilibrium regime.

## Materials and Methods

### Simulation

To gain intuition on the dynamics of populations under strong selection and migration, we performed stochastic simulations. Two populations of equal size *N* were instantiated with no standing genetic variation, under the assumption that beneficial mutations are costly before the change in environment.

Each generation, four steps are simulated in each population to adjust the counts of the alleles in populations A and B respectively, $\bar{A}$ and $\bar{B}$:

#### Mutation:

Each population draws a Poisson distributed number of mutations (λ=N×μ, where $\mu =10^{-5}$ (Abram *et al.* 2010)) which land on wildtype backgrounds and each create new, fully identified genetic lineages. All beneficial mutations confer identical fitness benefit 1+s relative to wildtype.

#### Selection:

Counts of different alleles in each population ($\bar{A}$ and $\bar{B}$) are normalized into frequencies $f_{A}$ and $f_{B}$, respectively. The frequency of each mutational lineage is deterministically adjusted based on its fitness benefit: the frequency of each non-WT allele is multiplied by 1+s. Then, all frequencies are normalized so the sum of all alleles within each population equals 1.

#### Migration:

Allele frequencies are deterministically adjusted based on the migration rate, *m*:

$$f_{A}^{\prime }=f_{A}\left(1-m\right)+f_{B}m$$

$$f_{B}^{\prime }=f_{B}\left(1-m\right)+f_{A}m.$$

#### Stochastic sampling:

*N* alleles each are sampled from $f_{A}^{\prime }$ and $f_{B}^{\prime }$ with replacement to produce counts for the next generation, $\bar{A}\prime$ and $\bar{B}\prime$.

### Approximate Bayesian computation

We employed approximate Bayesian computation (ABC) to estimate model parameters from simulated and real data (see below). We performed a two-step approximate Bayesian computation procedure in which we simulated $3\times 10^{6}$ forward trajectories with uniform $log_{10}$ priors where $m\in \left(10^{-5},5\times 10^{-1}\right)$, $s\in \left(10^{-1},10^{2}\right)$, and $N\mu \in \left(10^{-1},10^{1}\right)$. We restricted our Nμ prior to values expected to produce mutations that would then have time to sweep in the population within the relevant time frame of 100 generations.

We used rejection sampling, which accepts a certain percentage of trials (given by the tolerance) that minimize the Euclidean distance between the observed summary statistics and those summary statistics generated by the prior as implemented in the R package ‘abc’ (Csilléry *et al.* 2012). The parameter combinations that result in the lowest distances form the posterior. We first perform ABC to estimate the posteriors for Nμ and *s*, using targeted summary statistics as discussed in Aeschbacher *et al.* (2012).

For Nμ we use the best fit *θ* under Ewens’ Sampling Formula for when the populations are combined at each time point. Note, combining two population allele frequencies with limited migration overestimates the value of *θ* (*i.e.*, increases diversity relative to a single population), but this does not affect our procedure because the best fit *θ* is used only as a summary statistic.

To estimate *s*, we fit the observed frequency of the derived allele at the sampled time points to a logistic curve with y-intercept of $10^{-5}$ that minimized mean squared error from the observed data. In the cases of ties, we used the shallowest slope that could explain the data equally well.

From our first fit ABC, we get posteriors over Nμ and *s*. We then take the range of values within the 95% posterior for these two parameters and use those as a flat prior for a second fit (see Figure S12). For our second fit ABC, we use four summary statistics at each time point: $F_{ST}$, ${G^{\prime }}_{ST}$, the absolute value of the difference in heterozygosities between the two subpopulations, and the number of variants shared at any frequency. For a schematic illustration of the two-step process, see Figure S12. This two-step procedure drastically improved our estimates for *m* as compared to a one-step procedure (data not shown).

### Computation of summary statistics

Compartments *A* and *B* were sampled with allele counts for the *l* alleles at the beneficial locus $\bar{A}=\left(a_{1},a_{2},\ldots a_{l}\right)$ and $\bar{B}=\left(b_{1},b_{2},\ldots b_{l}\right)$. The total number of alleles sampled in each population was $\sum_{l}\left(a_{i}\right)=n_{A}$ and $\sum_{l}\left(b_{i}\right)=n_{B}$ with $n_{A}+n_{B}=n$. We use the $G_{ST}$ definition of $F_{ST}$, based on the difference between within- and between-compartment heterozygosities:

$$F_{ST}=\frac{h_{b}-h_{w}}{h_{b}}$$

where $h_{b}$ is the average within-compartment heterozygosity

$$h_{b}=\frac{n_{A}}{n}\left(1-{\sum_{i=1}^{l}\left(\frac{a_{i}}{n_{A}}\right)}^{2}\right)+\frac{n_{B}}{n}\left(1-{\sum_{i=1}^{l}\left(\frac{b_{i}}{n_{B}}\right)}^{2}\right)$$

and $h_{w}$ is the pooled-compartment heterozygosity

$$h_{w}=1-\sum_{i=1}^{l}\left(\frac{a_{i}}{n_{A}}\times \frac{b_{i}}{n_{B}}\right).$$

${G^{\prime }}_{ST}$ is a renormalized $F_{ST}$ based on the maximal value it can reach accounting for the most frequent allele in the population

$${G^{\prime }}_{ST}=\frac{F_{ST}}{Q(max\left(\sum_{i=1}^{l}\left(\frac{a_{i}+b_{i}}{n}\right)\right)}$$

where Q(f) is given in equations 15 and 16 of Jakobsson *et al.* (2013).

The difference in heterozygosities is computed as

$$\delta_{het}=\left|2-{\sum_{i=1}^{l}\left(\frac{a_{i}}{n_{A}}\right)}^{2}-{\sum_{i=1}^{l}\left(\frac{b_{i}}{n_{b}}\right)}^{2}\right|.$$

### Model evaluation

We determine whether the posteriors from ABC are providing useful estimates by comparing the posteriors to the true model parameter. The most useful posteriors will have small variance and be centered around the true values. However, using either one of these metrics will lead to misidentification. For example, if we evaluated a posterior using the proportion of the time the 95% posterior interval contains the true value, a completely uniform estimate across the posterior should contain the true value 100% of the time while offering no information about the estimated parameter. Alternatively, if we evaluated a posterior using the size of the 95% interval, a very narrow posterior far away from the true value would score highly but provide misleading information. To balance confidence and accuracy, we use the mean-squared error computed in log space (as our posteriors are uniform on a log-scale) to find the average difference between each of *n* points ($\sigma_{i}$) in our estimated posterior and our true test parameter *x*.

$$logMSE\left(\sigma ,x\right)=\frac{1}{n}{\sum_{i=1}^{n}\left(log_{10}\left(\sigma_{i}\right)-log_{10}\left(x\right)\right)}^{2}$$

### Simian-HIV data

To explore a structured population with strong selection and migration, we analyze macaque T98133 from Feder *et al.* (2017). Briefly, a macaque was infected with RT-SHIVmne027, a simian immunodeficiency virus with an HIV-1 reverse transcriptase region and treated with FTC, a reverse transcriptase inhibitor. Drug resistance mutations to FTC occur within the reverse transcriptase region. The viral population was given 12 weeks to establish within the host before treatment began. Viral samples were collected from the gut, lymph node and blood plasma at 1, 3, 8 and 14 weeks after the onset of treatment. The authors performed single genome sequencing of the reverse transcriptase region, resulting in 800 bp regions containing relevant drug resistance mutations and complete linkage information and have approximately 30 sequences per time point and location.

Generations in HIV/SHIV are approximately 24 hr, so we translated weeks 1, 3, 8 and 14 to generations 7, 21, 56 and 98, which we rounded to generations 5, 20, 50 and 100.

**Figure 1:**
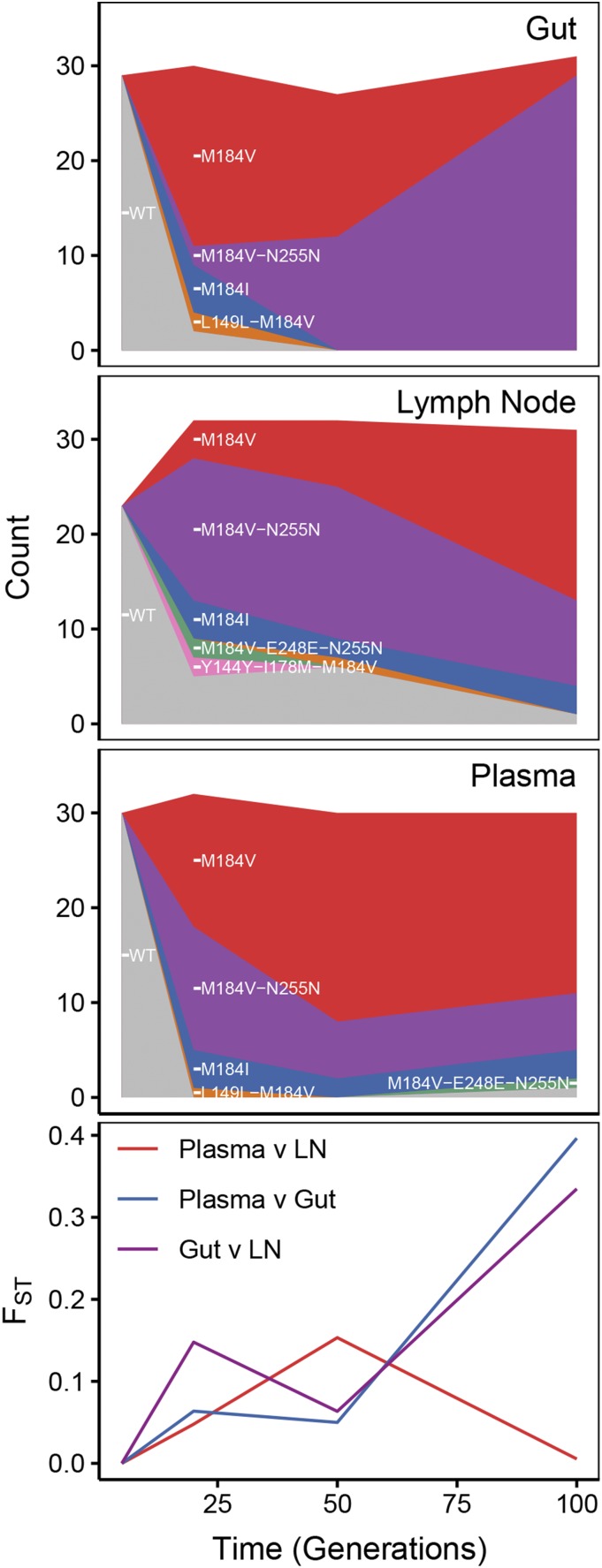
Dynamics of drug resistance fixation across space and time in a treated Simian-HIV population. The top three rows shows diagrams of drug resistant haplotypes spreading in different sampling locations over time sampled at generations 7, 21, 49 and 98 after the onset of selection via the drug FTC in the gut, lymph node and blood plasma. Each color represents a distinct lineage separated by at least one mutation. The bottom-most panel plots pairwise $F_{ST}$ between pairs of sampling locations.

We called derived haplotypes as unique if they contained a drug resistance mutation (M184V/I) and any other mutations at the initial time point at which drug resistance was observed (approximately generation 20) in at least c=2 copies. Note, we replot Figures 1 and 7 with choices of c=1 and c=5 in Figures S8, S9, S10 and S11. 95% posteriors across the choices of *c* are also listed in Table S1. We excluded haplotypes appearing for the first time after generation 20, to avoid mutations occurring on the backgrounds of existing lineages, which our model does not consider. These later time point haplotypes were clustered back to the most common early time point haplotype with the minimal mutational distance. For example, if generation 20 contained three haplotypes with the mutations A, B and C: A, A+B and A+C, and the haplotype A+B+D was observed at generation 50, it was counted as haplotype A+B.

No additional known drug resistance mutations appeared after generation 20, although positive selection may still influence allele frequency trajectories.

When we compute $F_{ST}$ pairwise between anatomical compartments (as in Figure 1D), we treat each haplotype as equally different from every other haplotype, despite some sharing more alleles than others. For example, M184I and M184V+N255N+D177N and WT are all equally different from each other. We do this in line with our single mutation simulation model, in which all haplotypes are equally different from each other. Computing differentiation statistics that take into account conservation does not qualitatively change the patterns, and this question is considered in much more detail in Feder *et al.* (2017). This convention is also used in the computation of all other summary statistics.

### Data availability

All code to reproduce the analyses can be found at https://github.com/affeder/fst_dynamics, and in the supplemental material. Sequences can be found in Genbank under accession numbers MF029756-MF030224 (plasma viral RNA) and MF032109-MF033066 (tissue viral RNA) for macaque T98133. Supplemental material available at FigShare: https://doi.org/10.25387/g3.9738875.

## Results and Discussion

### A motivating example using Simian-HIV drug resistance evolution

Viruses infecting a multicellular host may be subject to both strong selection (via drugs and the immune system) and also fast migration between anatomical compartments (via the circulatory and/or lymphatic systems).

A previous study found that among Simian-HIV within drug-treated macaques, multiple drug resistance mutations spread simultaneously across anatomical compartments with weak but significant evidence of population structure dynamically changing over time (Feder *et al.* 2017). In Figure 1, we reproduce a simplified picture of this evolutionary process in one Simian-HIV-infected macaque (T98133) across three well-sampled tissues (lymph node, gut and plasma) at four time points (1, 3, 8 and 14 weeks after the onset of selection via the drug FTC). Approximately 30 single-genome sequences of the reverse transcriptase region of the pol gene are taken per time point, per sampling location.

Each population has several different variants of drug resistant viruses spreading simultaneously which can be seen both through different encodings of drug resistant types (*i.e.*, M184V *vs.* M184I) and also through linkage to different hitchhiking mutations (M184V *vs.* M184V + N255N + D177N) (Figure 1). All colors shown in Figure 1 are resistant. Multiple spreading mutations suggest that the population mutation rate (Nμ) is sufficiently large so as to produce soft selective sweeps (Hermisson and Pennings 2005). These mutations quickly displace wildtype virus across tissues almost entirely, confirming that selection to drug resistance is strong. Finally, migration is sufficiently fast to spread the same mutations around to different compartments. While some of these apparently spreading drug resistance mutants may be recurrent mutations, several are linked to multiple putatively neutral mutations, suggesting that they arose once and then moved between anatomical compartments. However, migration is not so fast that each compartment looks equivalent, as demonstrated by statistically significant elevated pairwise $F_{ST}$ between compartments at some of the points during the experiment (Figure 1D) (see Materials and Methods for details, and Feder *et al.* (2017) for a much more extensive description of population differentiation in this data). Notably, we observe non-monotonic patterns of $F_{ST}$ with respect to time, suggesting that some pairs of populations differentiate as they fix beneficial mutations, then re-equilibrate over time (plasma v. lymph node and lymph node v. gut).

These patterns suggest qualitatively that the population genetic forces of mutation, migration and selection are all jointly strong. However, it remains quantitatively unclear how strong they are relative to each other. We therefore explore the dynamics of populations over short periods of time (including non-monotonicity of $F_{ST}$ relative to time) to better understand quantitatively how these population genetic parameters interact. We use many of the sampling attributes from the Simian-HIV example (≈100 generations, sampling depth of ≈30 genomes, ≈4 time points) as our reference in exploring this parameter space.

Although we use these data as a motivating example, many ecologically important processes feature rapidly adapting and dynamically interconnected populations.

### Building intuition about the interaction between strong selection and migration

We next explore how rapid adaptation in two subpopulations connected with migration can lead to signals of population differentiation over time.

**Figure 2:**
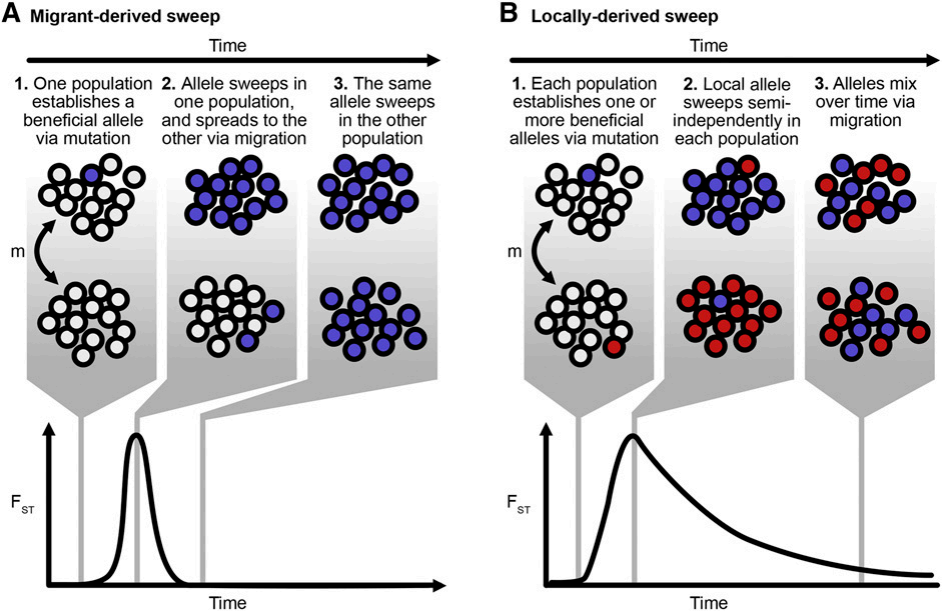
Schematic of allele frequencies and $F_{ST}$ across populations adapting in parallel. A. In a migrant-derived sweep, one population generates a beneficial allele via mutation significantly before the other population. The allele sweeps locally in its population of origin, and arrives in the alternative population due to migration before this population can fix its own variant. Here, the increase in $F_{ST}$ is due to divergent frequencies of the wildtype and derived allele, with the alternative population having a high frequency of the wildtype allele while the focal subpopulation has a high frequency of the derived allele. Migrant-derived sweeps have a short, symmetric spike in $F_{ST}$. B. In a locally-derived sweep, each population fixes its own variant (shown in red and blue) which increases in frequency locally. Over time, migration equilibrates the allele frequencies among the two populations. The increase in $F_{ST}$ is due to divergent frequencies among different derived alleles across populations, and $F_{ST}$ has a long-tailed, asymmetric trajectory over time.

Figure 2 shows two schematic scenarios through which a population divided into two subpopulations can fix beneficial variants. In both instances, a beneficial mutation will enter one population and begin to spread. However, the paths differ according to whether the first beneficial allele in the second subpopulation arrives through *de novo* mutation or via migration. Understanding differences in static signatures resulting from these two scenarios (in addition to selection from shared standing genetic variation between the two populations) are considered in Lee and Coop (2017).

In a “migrant-derived sweep” (Figure 2A), a beneficial mutation sweeps in one subpopulation and then spreads to the second subpopulation via migration. The mutation then sweeps in the second subpopulation. Population differentiation increases due to different frequencies of the same allele (or alleles) across subpopulations, and then disappears when the sweep completes in the second subpopulation. This case, which has been described previously in Slatkin and Wiehe (1998), Kim and Maruki (2011) and Bierne (2010), is more likely when the influx of beneficial mutations is limited (*i.e.*, Nμ low).

When the population mutation rate is high, another path is also possible (Figure 2B). If many beneficial mutations enter the population each generation, then each subpopulation may produce its own beneficial variant (Figure 2B1.) which begins to rise in frequency locally (Figure 2B2). Population differentiation (as measured by $F_{ST}$) increases due to sweeps of different alleles concurrently. However, over time, migration equilibrates the allele frequencies across the two populations (Figure 2B3), and $F_{ST}$ returns to long-term equilibrium levels. As we will explore later, the initial increase in differentiation and the rate of equilibration can be informative of the speed of migration.

The shape of the $F_{ST}$ trajectory over time differs substantially between the two cases. In locally-derived sweeps, selective sweeps drive the initial increase of $F_{ST}$ and migration erodes population differentiation over time. In the case of the migrant-derived sweeps, selective sweeps drive both the increase and decrease in $F_{ST}$, because $F_{ST}$ decreases as the sweep completes in the second subpopulation (See Figure 2A). Migrant-derived sweeps are therefore substantially less informative than locally-derived sweeps about the rate of migration between two populations, because both the increase and decrease of $F_{ST}$ are on the timescale of selection.

Whether a sweep will be locally- or migrant-derived is closely related to the probability of hard or soft sweeps (see Pennings and Hermisson (2006)), and a full exploration of the conditions is left to a future study. Because Kim and Maruki (2011) describe migrant-derived sweep behavior in detail, we focus on locally-derived sweep behavior, although both types of sweeps occur in the model described below. Further, we believe a model of locally-derived beneficial mutations represents the more probable description of the dynamics in our motivating example of SHIV.

#### Model:

We consider a two-population island model with mutation, migration and selection. We model two haploid populations of size *N* with non-overlapping generations. Each population begins fixed with an identical wildtype allele (standing genetic variation is discussed below). Each generation, mutation introduces new beneficial mutations at a rate Nμ at a single locus. Throughout the text, we focus on values of Nμ where mutations are readily available to the population (Nμ≥0.1, and often Nμ=1. Beneficial mutations possess an enhanced survival likelihood relative to wildtype ($w_{WT}=1$, $w_{b}=1+s$, s>0, see Materials and Methods for full details). All beneficial mutations are neutral with respect to each other, and no individual can carry multiple mutations. This describes, for example, loss of function mutations where subsequent mutations in the same pathway have no effect and follows the modeling assumptions of Ralph and Coop (2010). Each generation, a proportion of the population *m* migrates symmetrically between the two subpopulations. Therefore, Nm=M individuals migrate in each population per generation.

We investigate first the conditions under which the subpopulations will differentiate, as measured by the population differentiation statistic $F_{ST}$.

**Figure 3:**
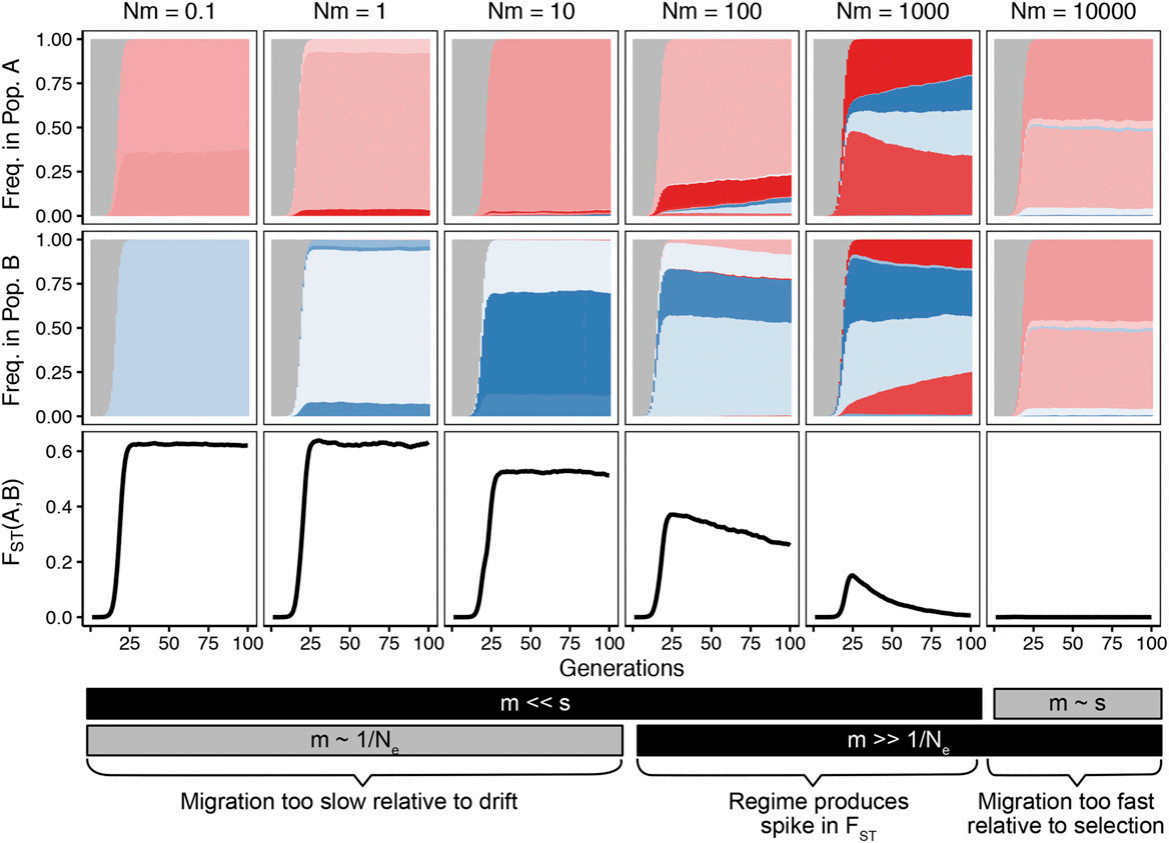
Patterns of allele frequencies and $F_{ST}$ across populations adapting in parallel. Beneficial alleles originating in populations A and B (colored red and blue respectively) increase in frequency over the wildtype allele (in gray) creating patterns of differentiation in $F_{ST}$ shown on the bottom row. The patterns of differentiation are dependent on the migration rate connecting the two populations (M=Nm shown in columns, $s=1,N=10^{5},m$ varies as listed in column header.)

Figure 3 shows a collection of stochastic simulations generated with Nμ=1. Each colored region represents the spread of an allele in the two large populations experiencing strong positive selection ($N=10^{5}$, s=1). Red alleles originate in population A and blue alleles originate in population B. When the migration rate is low, each population fixes independently its own set of alleles (see $M=10^{-1}$ through M=100). When the migration rate is high, the populations are indistinguishable and represent a mix of alleles originating in both subpopulations (*i.e.*, $M=10^{4}$). Among intermediate migration rates, however, more complicated dynamics arise. The population differentiation statistic $F_{ST}$ initially rapidly increases and then slowly decreases. The non-monotonicity of these trajectories in time can be understood by considering three relevant timescales: 1) the relative forces of selection and migration during the sweep, 2) the relative forces of migration and drift following fixation, and 3) the relative timescales of migration and the sampling.

The initial dynamics are determined by comparing the forces of migration and selection during the sweep. If selection overwhelms migration (s≫m), each population at least partially fixes its own variant and $F_{ST}$ increases rapidly (here, $M\leq 10^{3}$). Alleles spread within subpopulations faster than migration equilibrates these alleles across subpopulations. However, if migration is too fast compared to selection, little differentiation occurs at any point in the sweep ($M=10^{4}$).

The subsequent decrease in $F_{ST}$ depends on the relative timescales of migration and both genetic drift and sampling post-sweep. In our model, all alleles share identical selective benefit and no secondary mutations occur on the background of the first mutation (although the first mutation can happen on the wildtype background multiple times). Therefore, after loss of the wildtype allele, all alleles are selectively neutral with respect to each other, and solely migration and drift govern the changes in their frequencies. $F_{ST}$ between the two populations will ultimately decrease to near the equilibrium predicted by Wright ($F_{ST}=1/\left(1+4Nm\right)$), but the rate of that equilibration depends on the migration. If migration is sufficiently fast compared to drift (*i.e.*, $m\gg 1/N_{e}$), $F_{ST}$ will decrease faster than drift will move alleles within subpopulations. However, if migration is comparable to drift, the process will take significantly longer (on the order of $N_{e}$ generations). Whether this decrease is observed depends on the time frame of population sampling, although this paper will be restricted to relatively short sampling periods (*i.e.*, 100 generations).

These observations suggest that in adapting populations, distinctive patterns emerge depending on the relative values of migration (*m*), selection (*s*), genetic drift ($1/N_{e}$) and the sampling timescale. By examining these patterns over short timescales in genetic data, we may be able to estimate the values of *s*, *m* and *N* relative to each other in natural populations. This estimation will be a primary goal of the paper.

An analog of Figure 3 with a lower population mutation rate (Nμ=0.5) is shown in Figure S1, and displays example patterns of $F_{ST}$ in parameter regimes where migrant-derived sweeps are prevalent. If a similar picture is produced using neutral alleles, only the lowest migration rates produce differentiable patterns and elevated $F_{ST}$ (Figure S2), and it takes much longer for this differentiation to accrue (*i.e.*, on the order of $N_{e}$ generations).

The dynamic patterns of $F_{ST}$ under strong selection and migration suggest sweeping alleles across populations can provide insight into migration that occurs over much faster timescales than are relevant under neutrality. As in the neutral case, when migration is too slow compared to the rate of change in the population we can only say that migration rate belongs to a particular, slow regime. We encounter a similar situation when migration is too fast compared to selection. However, in the intermediate cases when selection and migration are on sufficiently similar timescales, more precise estimation appears possible. By observing alleles moving at different speeds due to different strengths of selection, we can move the boundaries of the bins and estimate more precisely migration at different speeds.

This example also illustrates several additional practical points. 1) Because dynamical patterns in $F_{ST}$ are apparent when sweeping alleles are captured at multiple points in their trajectories, this approach depends on the availability of time series data. 2) Because differences in the allele frequencies must be measurably different, sampling depth will affect whether or not these signatures are present.

Before dynamical patterns of population differentiation can be used to perform parameter estimation in a stochastic model, we first examine patterns of $F_{ST}$ under strong migration and selection in a deterministic setting. Specifically, we will explore analytically the parameter regimes that create transient and long-term population differentiation driven by locally-derived sweeps. We will see later that the population parameter regimes that create non-monotonic signatures of population differentiation with respect to time correspond to those in which we can successfully perform parameter estimation using approximate Bayesian computation.

#### Analytical approximation:

To study locally-derived sweeps, we consider the case in which each of two populations has a single copy of a local allele at the same locus (*i.e.*, frequency  f(0)=1/N) simultaneously. We ignore all subsequent mutation. Because the populations are symmetric, without loss of generality, we can consider the frequencies of a single subpopulation and determine what are the relative frequencies of the local and non-local allele at a time *t* ($f_{l}\left(t\right)$ and $f_{nl}\left(t\right)$, respectively). We consider the total number of derived alleles $f\left(t\right)=f_{nl}\left(t\right)+f_{l}\left(t\right)$ and assume that f(t) grows logistically.

$$f\left(t\right)=\frac{e^{st}}{e^{st}+1/f\left(0\right)-1}.$$

A differential equation describes the change in frequency of the non-local allele, $f_{nl}\left(t\right)$:

$${f^{\prime }}_{nl}\left(t\right)=m\left(f_{l}\left(t\right)-f_{nl}\left(t\right)\right)+sf_{nl}\left(t\right)\left(1-f\left(t\right)\right).$$

We define $F\left(t\right)=f_{nl}\left(t\right)/f\left(t\right)$, the frequency of the non-local allele among all derived alleles. We solve to find

$$F\left(t\right)=0.5+\left(F\left(0\right)-0.5\right)e^{-2mt}.$$ (1)

Closed form descriptions of $f_{nl}\left(t\right)$ and $f_{l}\left(t\right)$ are given as follows:

$$\begin{array}{l} f_{nl}\left(t\right)=f\left(t\right)F\left(t\right)\\ f_{l}\left(t\right)=f\left(t\right)\left(1-F\left(t\right)\right). \end{array}$$ (2)

We can investigate patterns of population differentiation using the $G_{ST}$ definition of $F_{ST}$, representing the scaled relationship between intra- and inter-population heterozygosity (see Materials and Methods). If we assume that each population starts with its own allele at arbitrarily low frequency (*i.e.*, $f_{l}\left(0\right)=1/N$ and $f_{nl}\left(0\right)=0$), we can describe the dynamics of the two alleles over time via via the following equation (see supplemental text for more explicit derivation):

$$F_{ST}\left(t\right)=\frac{{\left(1-2F\left(t\right)\right)}^{2}\;f\left(t\right)}{2-2\;f\left(t\right)F\left(t\right)\left(1-F\left(t\right)\right)-f\left(t\right)}.$$ (3)

**Figure 4:**
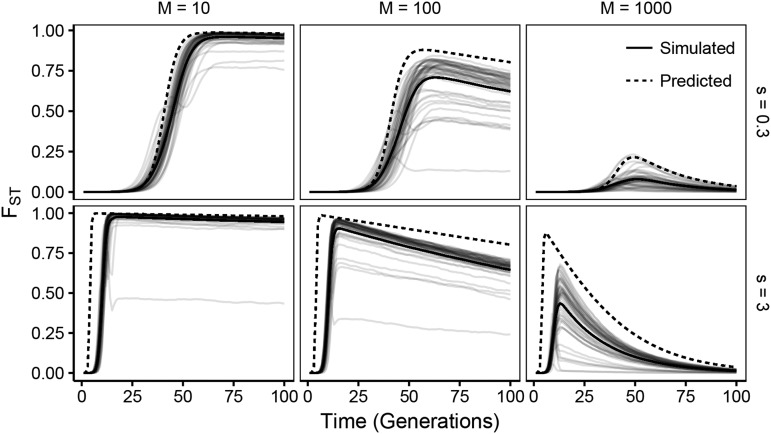
Simulated patterns of $F_{ST}$ over time share features with analytical predictions. Forward stochastic simulations of $F_{ST}$ between two populations undergoing parallel selection of strength *s* and connected by *M* variants per generation for a given set of parameters are shown in light, solid lines (100 replicates). All alleles originating in a single subpopulation are collapsed together for the purpose of computing $F_{ST}$. The median trajectory is shown in a dark solid line. Analytical predictions (equation 3) are shown in a dashed line. (Nμ=1).

The predicted $F_{ST}$ trajectories match well the trajectories generated by simulations showing locally-derived sweeps (Figure 4). Because multiple circulating alleles constrain the possible values of $F_{ST}$ (Jakobsson *et al.* 2013), all alleles originating from the same population from simulation are collapsed into a single allele so that the $F_{ST}$ magnitudes are also comparable.

This equation also allows us to quantify the generation at which $F_{ST}$ will be maximized between the two subpopulations ($t_{max}$), and its maximum value ($F_{ST}\left(t_{max}\right))$:

$$t_{max}=\frac{1}{s}\text{log}\left(\left(\frac{s}{m}-4\right)\left(N-1\right)\right)$$ (4)

and

$$F_{ST}\left(t_{max}\right)=\frac{2{\left(\frac{s}{m}-4\right)}^{1-\frac{4m}{s}}}{{\left(\frac{s}{m}-4\right)}^{1-\frac{4m}{s}}+\frac{s}{m}{\left(N-1\right)}^{\frac{4m}{s}}}.$$ (5)

From these equations, we can determine that the waiting time until the population reaches maximal $F_{ST}$ is dominated by a term inversely proportional to s. However, the maximal $F_{ST}$ at this point is determined by the ratio of s/m. The strength of selection controls largely when the $F_{ST}$ spike will happen, but the ratio of s/m governs the magnitude of the signal, with stronger selection relative to migration creating more pronounced spikes as a non-linear function of that proportion. Note that this equation is undefined when selection is not sufficiently stronger than migration (s<4m).

Below we will also use these equations to quantify the parameter regimes in which $F_{ST}$ will show non-monotonic patterns with respect to time by evaluating when $F_{ST}\left(t_{max}\right)$ will be elevated sufficiently for it to be observed at a given depth, and also when $F_{ST}$ will decline appreciably over a relevant timescale by evaluating equation 3 some number of generations after $t_{max}$.

Note that because this analytical framework assumes the simultaneous appearance of low frequency resistance mutations across populations, it only describes the dynamics seen when populations are non-mutation limited (*i.e.*, Nμ large). Some of the discrepancies between the predicted and simulated trajectories stem from violation of this symmetry in simulations.

### Patterns of population differentiation can be used for parameter estimation using approximate Bayesian computation

To translate these expected patterns into parameter estimates, we employ approximate Bayesian computation (ABC) (Rubin 1984; Beaumont *et al.* 2002) because exact likelihood formulations cannot be worked out under complex demographic scenarios. This provides a framework for interpreting allele frequency trajectories in the context of underlying parameter regimes. Briefly, ABC works through simulating data under parameters drawn from a prior and then comparing the simulated data to observed data. The parameter combinations that generate simulated data most similar to the observed data form posterior distributions. Note, we do not intend this as a claim for the best way to estimate migration using selected alleles, but as a demonstration of extractable signal.

We begin by demonstrating that migration information can be estimated from observed allele frequencies generated from our stochastic simulations using allele frequency differences in these connected populations over time. Recently, many methods have built up around the idea of estimating selection strength from allele frequency data in a single population (Bollback *et al.* 2008); Feder *et al.* 2014; Terhorst *et al.* 2015; Schraiber *et al.* 2016; Iranmehr *et al.* 2017). By leveraging this information across multiple populations, we can learn about selection and migration simultaneously. In particular, we imagine that we have allele frequency samples of *n* genomes before and after a sweep, and at some time point *T* generations later (here: n=100, T=30). These time points are chosen to match the Simian-HIV motivating example, but we provide a much more thorough analysis of how sampling time affects this approach below.

Because we are tracking the frequencies of many alleles across multiple time points, we use summary statistics to characterize the observed dynamics. As noted in Aeschbacher *et al.* (2012), the accuracy of ABC can be boosted when summary statistics are optimized separately for estimating different parameters, especially when there are only weak interactions between pairs of parameters. To jointly estimate *s*, *N* and *m*, we selected summary statistics that were useful for fitting the migration rate separately from *s* and *N*. We first performed a single round of ABC to estimate posteriors over *s* and *N* with these summaries. Then, we performed a second round of ABC to estimate *m* while restricting the priors over *s* and *N* to the posteriors from the first round fit (see Materials and Methods). We also fit *s* and *N* separately from each other and did not find a significant improvement in method performance (data not shown). To summarize information about *N*, we found the most likely *θ* using Ewens’ Sampling Formula for the combined allele frequencies at each sampled time point pooled across the populations. To summarize information about *s*, we assumed beneficial allele frequency $10^{-5}$ for a beneficial mutation at time 0 (which is a conservatively low frequency for intra-host standing genetic variation (Zanini *et al.* 2017; Theys *et al.* 2018)) and determined the parameter *s* producing the logistic growth curve that minimized mean squared error from the observed data. To compute *M*, we used four parameters at each time point: $F_{ST}$, ${G^{\prime }}_{ST}$, the difference in heterozygosities between the two subpopulations and the number of shared alleles at any frequency. Reasoning and further details concerning these summary statistics can be found in the Materials and Methods.

#### Migration estimation depends on s:

**Figure 5:**
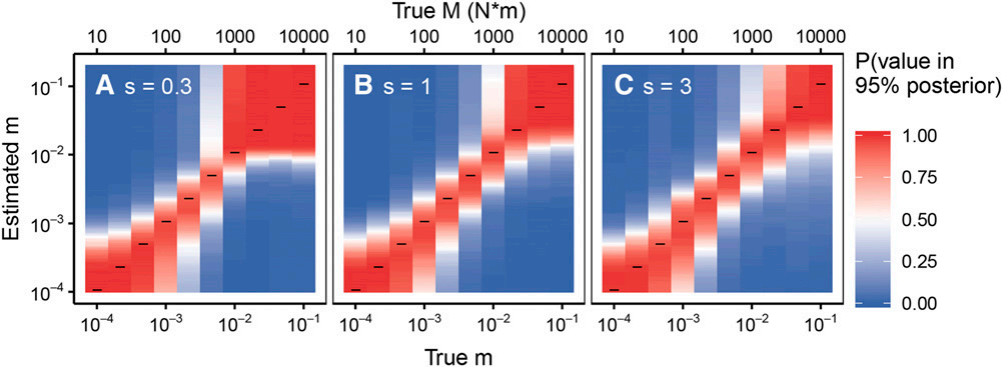
ABC procedure re-estimates true *M* values in simulated data. 200 simulations are run for each true value of *m* on the *x*-axis and 200 posteriors are produced. We plot the proportion of 95% posteriors for a given true *m* (shown on the *x*-axis) that include the various values of estimated *m* (shown on the *y*-axis). Values that are more red indicate that the posteriors successfully capture the test ‘Estimated *m*’ value on the *y*-axis whereas blue values indicate that few posteriors include the *y*-axis value. Black horizontal lines indicate where the true value matches the *y*-axis ‘Estimated *m*.’ Simulations are generated across a gradient of values for *m* and *s* ($m\in \left(10^{-4}-10^{-1}\right),s=\left(0.3,1,3\right)$, sampling at generations (5, *T* = fixation, $T+30,100),\mathrm{N=}10^{5})$.

To validate our ABC approach, we first simulated data under known parameters and then used our approach to re-estimate migration rates. We ran 200 forward simulations for each set of parameters and examined the resulting 95% posteriors over *m* to quantify the proportion of posteriors that contain the true *m* (Figure 5). See Materials and Methods for full simulation details. We also quantify the probability that the posteriors contain incorrect values of *m* at varying distances away from the truth. We find the 95% posteriors contain the true migration rate with high probability (Figure 5, indicated by the clustering of red around the x=y line) and do not contain migration rates far away from the truth (indicated by off x=y).

There are instructive exceptions. First, when the migration rate is low, we have little power to distinguish values of *m* in this range. Low *m* results in near total differentiation between the two subpopulations (see Figure 3, M<1000), so it is therefore unsurprising that we cannot predict the migration rate beyond bounding it. Similarly, this method cannot differentiate among very high migration rates. In this instance, the populations are entirely panmictic, and there is little signal to uncover (See Figure 3, $M=10^{4}$). Although within these regimes where allele frequencies from the two subpopulations appear panmictic or independent we have no ability to estimate migration rates specifically, this does allow us to bound fast migration rates much more precisely than is possible with neutral alleles. For example, if we were to observe identical equilibrium neutral allele frequencies among two subpopulations, we might conclude that M>1. However, if we observed identical allele frequencies over time subject to strong selection, this would suggest that $M\geq 10^{4}$.

**Figure 6:**
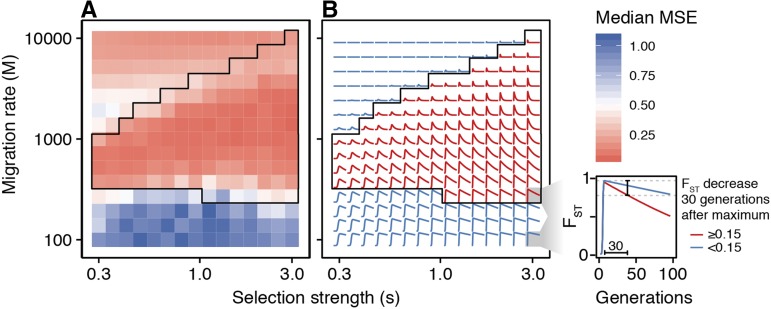
Better estimation of higher migration rates among simulations with higher selection strength. For a fixed *N*, higher *s* values result in lower log MSEs (indicating successful estimation) for higher values of *M*. Each tile represents the median log-MSE for 100 trials computed with a given *m* and *s*. ($N=10^{5}$, sampling at generations 2, *T* = fixation, T+30). B. Predictions of the dynamics of $F_{ST}$ over 100 generations under a variety of parameters for *s* and *m*. Each square in A. corresponds to the modeled dynamics in B. The parameter combinations predicted to lead to a 0.15 unit decrease in $F_{ST}$ after 30 generations (red) correspond to the parameter regimes where migration can be estimated with low MSE. The area circumscribed in black highlights this region in both A and B.

The boundaries at which we enter these regimes of independence and panmixia depend on the strength of selection. We see that s=0.3,M=1000 results in apparent panmixia in many instances. However, when s=3, our method still accurately estimates migration rates when M=1000 (Figure 5). We test this more directly by computing the log MSE between the posteriors and the true *m* for a wide variety of selection strengths and migration rates (Figure 6A, see Materials and Methods for a detailed description of the log MSE). Consistent with the analysis in Figure 5, when migration is too high or too low relative to selection, the posterior is far from the truth. (Note, the relatively low MSE between the truth and the posterior among high values of *m* is driven by the limits on the prior, which is bounded above by m=0.5). However, the upper boundary at which the performance deteriorates increases with selection strength. Put another way, as selection strength increases, so do the rates of *m* that can be accurately estimated.

These results are predicted by our analytical model of locally-derived sweeps. The parameter combinations that show a noticeable non-monotonic pattern in $F_{ST}$ over 100 generations (*i.e.*, $F_{ST}$ reaches a value greater than or equal to 0.15 followed by a decrease in $F_{ST}>0.15$ within 30 generations of $t_{max}$) are similar to those that can be estimated using the ABC approach (Figure 6B). This suggests that a primary signal identified by our ABC approach is a non-monotonic pattern of $F_{ST}$ over time. However, because the performance of the ABC approach deteriorates as other summary statistics are removed (data not shown), this is likely just one of several important signals for estimation.

### Practical considerations in estimating m from data

The interaction between migration and selection is not the only factor that impacts our estimation. We find that the performance improves when the sampling is deeper, reflecting more accurate estimation of allele frequency differences (Figure S3). We also find that the choice of sampling time points matters for the estimation of specific migration rates. We assume that samples are taken before and after a sweep, and then at an additional time point *T* generations later. When *T* is small, performance is worse, presumably because migration has had less time to influence allele frequencies (Figure S4). When adding a fourth time point at generation 100, the method is fairly insensitive to the placement of the third time point (Figure S5).

We also find that specific parameters of the ABC are important, namely the tolerance of accepted matches. We find that as tolerance becomes more stringent, the posteriors become narrower (Figure S6A). However, as the resulting posterior decreases in size due to lower tolerance, there is a decreased probability of capturing the true value in the posterior (Figure S6B). Nevertheless, decreasing the tolerance improves the matching of posteriors and the truth (Figure S6C), and we therefore choose a low tolerance (tol=0.001) for our analyses.

Finally, we explore how true asymmetries in the underlying model affect ABC performance in estimating migration rates (Figure S7). Similar to analyses above, we simulate adaptation to strong selection ($N_{A}=10^{5},s_{A}=1$) across two subpopulations under a variety of migration rates, but now we allow the second subpopulation to differ in migration rate ($m_{AB}=pm_{BA},p\in \left(0.1,0.5,1\right)$, Fig S7A-C), population size ($N_{A}=pN_{B},p\in \left(0.1,0.5,1\right)$, Fig S7D-F) or selection strength ($s_{A}=ps_{B},p\in \left(0.1,0.5,1\right),$ Fig S7G-I). We estimate migration rates assuming no asymmetries.

Our model is relatively robust to differences among migration rates between the two subpopulations ($m_{AB}\neq m_{BA}$). When $m_{AB}\leq m_{BA}$, the estimated migration rate is intermediate to the two rates, but has similar variance to the symmetric case (Figure S7A-C).

Differences in population size between the two subpopulations increase the variance of the posteriors, but do not appear to bias the estimation of migration rates (Figure S7D-F).

Differences among selection coefficients between the subpopulations create more complicated effects. When selection in one subpopulation is much smaller than the other (but selecting for the same variants), we are unable to estimate migration because the sweep does not complete across both compartments, and our estimation procedure conditions on a near full sweep (Figure S7G). When migration is sufficiently fast, migration from the subpopulation experiencing stronger selection contributes substantially to the sweep in the subpopulation experiencing weaker selection. As a result, when the populations are sampled, they appear more similar to each other than if both populations are increasing alleles due to strong, semi-independent selection. As a result, migration rates are systematically biased upwards, but the effect is small (Figure S7G-H). In addition, because the selection strength is lower in one subpopulation, the method has less resolution to differentiate among intermediate migration rates, as we observed in the investigation above. This also leads to an increase in variance among the estimated rates. We discuss how this compares to the case in which selection selects for different variants across compartments in the discussion.

#### Application to real data:

In the previous section, we have established that we can estimate population genetic parameters using a multi-step ABC method. In this section, we apply this method to real data that matches the structure of our ABC method (Feder *et al.* 2017) in order to estimate viral migration rates between pairs of different organs (blood plasma, lymph nodes, gut) of a Simian-HIV infected pigtailed macaque sampled over time (Figure 1). The importance of estimating such rates has been previously investigated (although in the absence of selection) Ewing and Rodrigo (2007). The allele frequencies shown in Figure 1 are simplified trajectories of the full data to make it more comparable to our model. For full data processing choices (and fits when those choices are modified), see the Materials and Methods section.

**Figure 7:**
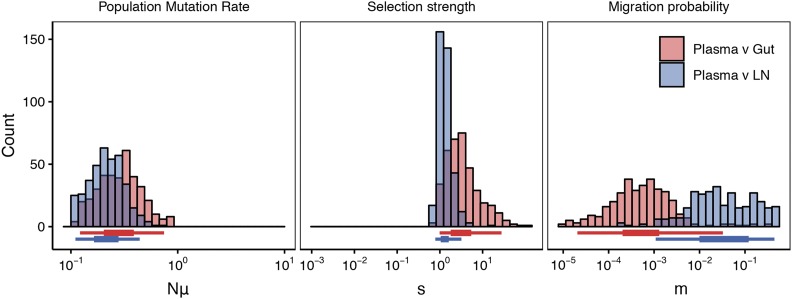
Estimation of population parameter rates from intra-patient Simian-HIV data sampled from different subpopulations. The top row shows diagrams of drug resistant haplotypes spreading in different subpopulations over time sampled at generations 7, 21, 49 and 98 in the gut, lymph node and blood plasma. Each color represents a distinct lineage separated by at least one mutation. The resulting posteriors are given for the ABC procedure for comparisons between the plasma and gut (red) and plasma and lymph node (blue). $tol=2.5\times 10^{-4}$. 95% posteriors for these distributions are shown in Table 1.

We find that estimates of *m* between the plasma and lymph node are very high: The distribution is flat above 1% of the population migrating each generation (Figure 7, Table 1). The flatness of this distribution suggests that these subpopulations exist in the regime in which migration moves alleles faster than selection, and we find little differentiation that cannot be explained by sampling error (consistent with findings of panmixia in Feder *et al.* (2017)). Although a more specific migration rate cannot be estimated, posteriors suggest a migration rate above m≥0.001.

**Table 1:** Medians and 95% posteriors for parameter estimation in intra-macaque Simian-HIV populations

	*m*	*M* *^a^*	*s*	Nμ
Plasma v Gut	5.28e-4	15.5	3	0.293
	(2.06e-5,0.0326)	(0.602,953)	(0.987,27.7)	(0.122,0.744)
Plasma v Lymph Node	2.71e-2	573	1.24	0.212
	(1.09e-3,0.444)	(23,9400)	(0.783,3.19)	(0.11,0.44)

a *M* is a composite estimate taken from multiplying the *m* distribution by the point estimate of predicted population size derived from the estimated Nμ (M=m×(Nμ)/μ).

However, the migration rate between the plasma and the gut is unimodal centered around 0.05% of the population migrating each generation (Figure 7, Table 1) - half of the low end of the posterior between the lymph node and the plasma, yet higher than what would produce elevated $F_{ST}$ in drifting alleles at equilibrium (although $N_{e}m=1$ falls within the 95% posterior). These differences between the connectivity between the plasma and gut or lymph node are biologically consistent in their ordering, as we might expect the circulatory system to be more connected to the lymphatic system than a mucosal tissue.

We estimate similar selection strength and population mutation rates across subpopulation comparisons (see Table 1). Across both comparisons, the selection strength is extremely strong (s≈1.24 - 3), which is necessary to explain the almost complete sweeps observed in the first 20 generations. The population mutation rate is sufficiently high that soft sweeps are likely (Hermisson and Pennings 2017). That we see no significant differences between the estimated population sizes makes intuitive sense given the consistency of sweep timing and diversity across the three populations. However, it is also possible that the size estimation across the pairs of compartments is driven by the size of the largest subpopulation, resulting in comparisons that are less dependent on the size of the smaller subpopulation. This would decrease our power to detect differences in compartment sizes in comparisons across ABC runs.

Note, we make filtering choices to determine what is counted as a distinct haplotype (see Materials and Methods for full details). In the main text, we present intermediate filtering in which we create haplotypes from alleles observed at least twice before drug resistance sweeps (>70%) (*i.e.*, alleles that are singletons before fixation are excluded). We can repeat the analysis for alleles observed in at least one copy (Figure S8) or at least five copies (Figure S9). Different haplotype thresholds unsurprisingly alter estimates of Nμ, but posteriors are robust for estimates of *s* and *m*, likely due to the factorization procedure (Figures S10 and S11). Posteriors for all three allele frequency cutoffs are listed in Table S1.

## Discussion

In this study, we characterize the dynamics of populations adapting under strong and uniform population genetic forces - abundant mutation, strong positive selection and fast migration between subpopulations. Specifically, we describe how beneficial mutations can spread locally within populations causing rapid population differentiation. Fast migration can then re-equilibrate the allele frequencies of the populations over short timescales. This can happen in two ways: either a beneficial mutation can sweep in one subpopulation and then establish in a second subpopulation via migration (migrant-derived sweeps), or each subpopulation can produce its own beneficial mutation(s) which ultimately equilibrate in frequency via migration (locally-derived sweeps).

Although migrant-derived sweeps have been described previously (Slatkin and Wiehe 1998; Kim and Maruki 2011; Bierne 2010), locally-derived sweeps can help explain the apparent “softening” of hard sweeps in HIV, which initially show only a single beneficial haplotype, yet later multiple haplotypes appear (Paulose *et al.* 2019; Ralph and Coop 2010; Hermisson and Pennings 2017; Williams and Pennings 2019). If each subpopulation fixes its own beneficial variant, which later mix, a locally hard sweep can appear to become soft. This is in contrast to the previously described “hardening” of soft sweeps, which can occur when demography causes only a single beneficial haplotype of a soft sweep to fix (Wilson *et al.* 2017).

That allele frequencies can change dynamically over very short timescales has important implications for interpreting population differentiation statistics. In particular, data sampled at a particular time point can be misleading in determining population parameters if the underlying population is incorrectly assumed to be at equilibrium. Even with constant migration rates, samples at different time points could lead one to conclude that migration is either prevalent or rare, and migration rates much larger than M=1 can lead to substantial population differentiation transiently. This suggests that merely observing elevated $F_{ST}$ is not a sufficient condition for diagnosing a low-migration system and understanding non-equilibrium dynamics of these statistics is important for analyzing data. Proper accounting for the covariance among selected alleles can help disentangle the signatures left by combined selection and migration (Lee and Coop 2017).

To understand the conditions in which populations appear differentiated over short timescales following a selective sweep, we explore an analytical model of $F_{ST}$ between populations with strong migration, selection and mutation. In time series data, we find a variety of dynamics of elevated $F_{ST}$: if selection is stronger than migration (s≫m), $F_{ST}$ will increase over short timescales. If migration is faster than drift ($m\gg 1/N_{e}$) and the sampling timescale, $F_{ST}$ will quickly decrease after the sweep. When both conditions are met, characteristic non-monotonic patterns of $F_{ST}$ emerge over experimentally tractable timescales. We introduce a modeling framework to further explore this diversity of patterns.

After observing a diversity of patterns in $F_{ST}$ as function of the relationships between drift, selection and migration, we develop an iterated-ABC framework that estimates *N*, *s* and *m* jointly using summaries of population differentiation over time. We explore the regimes where estimation is accurate, and find that its accuracy can be predicted from our simple analytical model of $F_{ST}$. In the parameter regimes where estimation is not accurate, we can bound migration rates above or below certain values by comparing the rate of migration to the processes of drift and selection. These broad regimes of estimation suggest that we can quantify migration rates that we might expect to be important among pathogen populations.

While the model-based portion of this analysis focuses mainly on one metric - $F_{ST}$ - to explore patterns of differentiation between connected populations, the inference framework also exploits other summarized aspects of the data including counts of shared haplotypes, and alternative normalizations of heterozygosity. While we do not here disentangle exactly what each summary statistic contributes, this does suggest that subtle differences in allele frequency trajectories encode information that can be useful for estimation. This underscores the potential for machine learning in population genetic inference Sheehan and Song (2016).

As an example of the inference approach, we estimate migration rates between viral populations inhabiting different organs in a Simian-HIV infected macaque treated with drugs. Consistent with the findings of the original study which used several metrics of population differentiation but did not attempt to estimate rates, we find differential connectivity between compartments of the body. In particular, we find that mucosal tissues (*i.e.*, the gut) have lower migration rates into the blood than the lymph nodes do. However, using our ABC framework, we can make this description of the intra-patient environment quantitative as opposed to simply qualitative. We estimate per-virus migration rates that can be used in future modeling studies.

One aim of this analysis was to explain a conspicuous pattern observed during the first ≈100 generations after the onset of selection in spatially-sampled simian-HIV populations - a rapid increase in $F_{ST}$ that decays quickly over time. In this paper, we show that some of the key features of this pattern can be explained in a simple symmetric island model. Notably, these key features appear without assumptions of asymmetry among compartments or local adaptation (similar to the observation that distinct patterns of sweeps across subpopulations can exist without local adaptation in Bierne (2010)). However, what is necessary is a migration rate of the right order of magnitude, conditional on the strength of selection.

Note, this does not imply that in the case of Simian-HIV the observed pattern cannot be produced in a different way. For example, selection strength or direction may differ among compartments caused by varying degrees of penetrance of antiretroviral drugs among organs of the body. If selection pressures differ between compartments (*i.e.*, a beneficial mutation in one compartment is neutral or even deleterious in the other), we are likely to underestimate migration rates, because allele frequencies will equilibrate more slowly between the two compartments, ultimately reaching migration-selection equilibrium. With strong and potentially temporally-varying local adaptation, we should be able to obtain the observed patterns with even much stronger migration.

However, since complex selection scenarios are not necessary to produce key features of our observations, we need not assume they are present. Accordingly, we believe that the mechanisms that are described by our model provide a plausible explanation for the initial dynamics of population differentiation. This said, there is some circumstantial evidence that over longer time scales selection differences could play a role. For example, by generation 98, a different clone comes to dominate the gut compartment than the lymph node and plasma. Indeed, fitness differences among subsequent selective sweeps (neither of which are incorporated in the model) can explain this phenomenon.

Other factors, such as asymmetries in mutation rates or compartment sizes could potentially lead to biases in the migration rates relative to our estimates (although likely to a lesser extent than differences in selection). Natural extensions to this model might incorporate differences in natural selection among populations, or include isolation-by-distance as opposed to a simple island model. An additional problem is that in non-mutation limited systems, not only do multiple mutations arise simultaneously, but populations quickly acquire double and triple mutants. It is therefore important that in future studies, we consider how these patterns of population differentiation might appear on a traveling wave of beneficial mutations, similar to what has been done in single populations (Desai and Fisher 2007).

Our results also provide guidance for experimental design, particularly in non-mutation limited systems with strong selection and potentially high migration rates such as pathogen evolution, and pesticide and herbicide resistance. In particular, we find that the most useful way to glean information about migration rates from data are to include a temporal component to sampling. For migration estimation, it is considerably more useful to have three time points of 30 sequences than a single time point of 90 sequences. It is particularly useful to sample during or shortly after a selective sweep. From a practical perspective, the timing of such sweeps can be determined by phenotypic shifts in the population. If only a single time point directly after the sweep is available, this can provide information about whether migration is slower than selection. However, if an additional later time point is available, this potentially also gives information about whether migration is faster than drift. Having samples at both of these times provides both upper and lower bounds for high migration rates.

To understand very rapid processes (such as fast migration) or the forces governing the population in its current state, it is insufficient to look at dynamics averaged out over long time periods. Selection provides an avenue to observe the current population state in time that neutral processes can miss. Using long term metrics (like comparing the rate of migration to the rate of drift) to investigate how much migration is happening in the moment invites numerous misinterpretations. When studying migration, as when studying all population processes, the correct timescale must be carefully considered.
